# Differences in Reversion of Resistance Mutations to Wild-Type under Structured Treatment Interruption and Related Increase in Replication Capacity

**DOI:** 10.1371/journal.pone.0014638

**Published:** 2011-01-31

**Authors:** Agnes C. Paquet, John Baxter, Jodi Weidler, Yolanda Lie, Jody Lawrence, Rose Kim, Michael Bates, Eoin Coakley, Colombe Chappey

**Affiliations:** 1 Monogram Biosciences, Inc., South San Francisco, California, United States of America; 2 Cooper University Hospital/UMDNJ-Robert Wood Johnson Medical School, Camden, New Jersey, United States of America; 3 University of California San Francisco, San Francisco, California, United States of America; University of Texas Medical Branch, United States of America

## Abstract

**Background:**

The CPCRA 064 study examined the effect of structured treatment interruption (STI) of up to 4 months followed by salvage treatment in patients failing therapy with multi-drug resistant HIV. We examined the relationship between the reversion rate of major reverse transcriptase (RT) resistance-associated mutations and change in viral replication capacity (RC). The dataset included 90 patients with RC and genotypic data from virus samples collected at 0 (baseline), 2 and 4 months of STI.

**Principal Findings:**

Rapid shift towards wild-type RC was observed during the first 2 months of STI. Median RC increased from 47.5% at baseline to 86.0% at 2 months and to 97.5% at 4 months. Between baseline and 2 months of STI, T215F had the fastest rate of reversion (41%) and the reversion of E44D and T69D was associated with the largest changes in RC. Among the most prevalent RT mutations, M184V had the fastest rate of reversion from baseline to 2 months (40%), and its reversion was associated with the largest increase in RC. Most rates of reversion increased between 2 months and 4 months, but the change in RC was more limited as it was already close to 100%. The highest frequency of concurrent reversion was found for L100I and K103N. Mutagenesis tree models showed that M184V, when present, was overall the first mutation to revert among all the RT mutations reported in the study.

**Conclusion:**

Longitudinal analysis of combined phenotypic and genotypic data during STI showed a large amount of variability in prevalence and reversion rates to wild-type codons among the RT resistance-associated mutations. The rate of reversion of these mutations may depend on the extent of RC increase as well as the co-occurring reversion of other mutations belonging to the same mutational pathway.

## Introduction

Treatment Interruptions (TI) can occur in clinical practice due to drug toxicity, patient non-adherence and antiretroviral treatment (ART) fatigue. In the setting of multi-drug resistant (MDR) viremia, the prevailing concept is that during treatment interruption, the MDR strain is rapidly overgrown by wild-type virus at higher HIV-1 RNA levels [1], associated with reversion of resistance mutations to wild-type codons, a shift toward phenotypic drug susceptibility, and an increase in viral replicative capacity, leading to the restoration of sensitivity to antiretroviral drugs [1]. With the re-introduction of ART, resistant strains genotypically similar to baseline virus may emerge [2], [3].

Currently, in clinics with ready access to the more recently approved antiretrovirals, the number of individuals with treatment associated viremia and multi-drug resistance is declining [4]. Further, treatment interruptions as therapeutic strategies have fallen out of favor because of the accelerated loss of CD4+ cells and nonHIV adverse events [5], [6]. However, there are a number of reasons to continue to explore TI in the setting of MDR. Firstly, despite the availability of newer therapies, there are concerns that the pipeline for future anti-HIV drug development has diminished. As a consequence, individuals with MDR in today's clinic may have few treatment options and for these individuals it is important to continue to explore alternative treatment strategies. Further, in resource limited settings, there may be few antiretroviral options, in which case maintaining therapy in the setting of drug resistance may be unavoidable. Also, transmitted drug resistance is of concern in both the adult patient and in the setting of mother to child prophylaxis (particularly in more resource limited settings). TI studies may provide a relevant perspective on the loss of mutations for individuals with transmitted drug resistance [7], [8].

Clinical outcomes in patients undergoing structured treatment interruption (STI), in which treatment is withdrawn for a fixed time period, have been described in a number of studies. However, the extent of reported genotypic changes during STI differs between studies, and only a few of these studies described the patterns of reversion of mutations to wild-type codons, which occur as a combination of re-emergence of preexisting variants with fewer resistance mutations and actual reversion of codons within a viral genome. An early study reported that almost all patients showed complete genotypic and phenotypic reversion of the dominant viral strain from multi-drug resistant to wild-type virus [1]. Longitudinal analysis of drug resistance in these samples showed that changes in the dominant viral strain occurred at various times, generally abruptly rather than progressively. Interestingly, fewer genotypic changes during STI were reported in a second study [3], as only 5 patients out of 18 showed complete reversion of mutations based on standard population-based sequences, suggesting inter-patient differences in the potential of reversion of dominant viral strains. Devereux et al [9] measured the decline of detectability of protease (PR) and reverse transcriptase (RT) resistance associated mutations in patients stopping therapy for varying durations of time. The authors reported a faster rate of reversion for primary resistance mutations (K70R, M184I/V, T215Y/F in RT, and D30N, M46I/L, V82A, L90M in PR) compared to secondary mutations (M41L, D67N, T69D/N, L210W, K219Q/E in RT and L10I/V, L63P, A71V/T, V77I in PR). Similarly, a stepwise pattern of reversion of resistance associated mutations (RAMs) to wild-type was described in one multi-drug resistant HIV-positive individual by comparative analyses of longitudinal population-based sequences [10]. A mixture of the mutant V and the wild-type variant M at amino acid 184 in RT was first detected in the MDR patient followed by complete reversion to the wild-type amino acid at this position. Mixtures of wild-type and variants T215Y in RT, L10F, I54V and V82A in PR appeared 6 months after the first mixture at position 184 was detected. Together, these studies suggest the possible co-existence of mechanisms of back-reversion and outgrowth of wild-type variants during STI, where the prevalence of the mutant viruses depends on virus fitness gain and other host factors.

Resistance mutations have been shown to accumulate in a specific order [11], but little is known about the dynamics of reversion to wild-type population. The CPCRA 064 study, the largest published randomized trial of STI in patients with MDR HIV-1, demonstrated that STI was not associated with virologic benefit but instead had a prolonged negative impact on CD4 counts [12], [13]. Changes in genotypic patterns were observed in the majority of individuals during STI, although some patients showed only partial or no shift in their viral population. The aim of this study was to describe the mechanisms of reversion of RT mutations to wild-type viral population during STI and the relationship with replication capacity (RC) as a measure of fitness. Using the longitudinal data collected at baseline, 2 months and 4 months of STI in the CPCRA 064 study, we will describe the patterns of loss of resistance-associated mutations and emergence of wild-type (WT) virus. We will identify early/late reverting mutations, linkage between mutations, and the impact of the reversion on viral RC, in an attempt to identify inter-patient differences in the rate of reversion to wild-type viral population.

## Materials and Methods

### Study dataset

The CPCRA 064 study examined the effect of 4 months of STI followed by salvage treatment in patients failing therapy with multi-drug resistant HIV. This study was approved by the institutional review board at all participating sites and all patients had written informed consent. Details regarding the study design of this trial and the list of all participating sites are described elsewhere [12], [13]. Of the 274 patients enrolled in the trial, 140 were randomized to the STI arm. 122 of those patients had sufficient sample available for retrospective replication capacity testing. Information regarding mutation score and replication capacity was collected at 0 (baseline), 2, and 4 months of STI. Not all patients were able to tolerate 4 months of STI; 32 patients resumed treatment before the end of the 4 month STI. This analysis was performed on the 90 patients who were able to sustain the 4 month STI. Among those 90 patients, 6 patients were further excluded from the mutation reversion analyses due to missing genotypic data at one of the 3 timepoints.

### Phenotypic and genotypic data

Viral replication capacity (RC) was retrospectively determined using a modified PhenoSense® HIV assay (Monogram Biosciences, South San Francisco). RC values were expressed as a percentage of NL4-3 drug-sensitive reference and adjusted so that the median value of wild-type viruses approximated 100%. Genotyping for the clinical trial was performed using the TruGene® 4.0 assay (Visible Genetics, Inc.). In the TruGene® assay, a 1.3-kb sequence from the pol region encompassing the entire protease gene and the first 250 amino acids of the reverse-transcriptase gene is generated by bidirectional automated sequencing on the Microgene Clipper (Siemens Diagnostics, Inc.). HIV-1 drug resistance mutations were identified using the October 2004 International AIDS Society definition [14]. Genotypic data consisted of the binary-coded presence/absence of 54 mutations in the reverse transcriptase (RT) and protease (PR) genes. Mutations present in fewer than 10% of the samples at baseline were not considered in the reversion rate and tree analyses. The final list of mutations included M41L, E44D, D67N, T69D, K70R, L74V, L100I, K103N, V108I, V118I, Y181C, M184V, G190A, L210W, T215F, T215Y and K219Q in RT; and L33F, L33I, M46I, M46L, G48V, V82A, V82F, V82L, V82S, I84A, I84C, L90M in PR.

### Statistical analyses

The significance of changes in RC over time was tested by Jonckheere-Terpstra trend test and Wilcoxon signed-rank test for paired samples. The rate of reversion of a mutation was estimated as the percentage of patients with a mutation reverting between 2 timepoints. The reversion rate was calculated from a subset of 84 patients who sustained the STI for at least 4 months and for whom genotypic data was available at baseline, 2 and 4 months. Concurrent reversion rate between two given mutations was calculated as the rate of simultaneous reversion of these 2 mutations between 2 consecutive timepoints (Equation 1). This co-reversion value was used as a marker of potential linkage between reverting mutations. 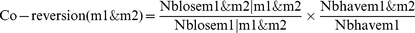
(1)


The order of reverting mutations was examined using mutagenetic tree models [15]. In our case of reversion events, tree vertices represent a reversion at a specific codon and edges are associated with the conditional probability that a reversion will occur, given that the ancestor in the tree has occurred. These models require non-reversibility of genetic events, which is verified in this dataset as none of the mutations changed back to a variant form from the wild-type sequence over the course of the STI. We used the software package Mtreemix [16].

## Results

### RC change during STI

**Figure 1:**
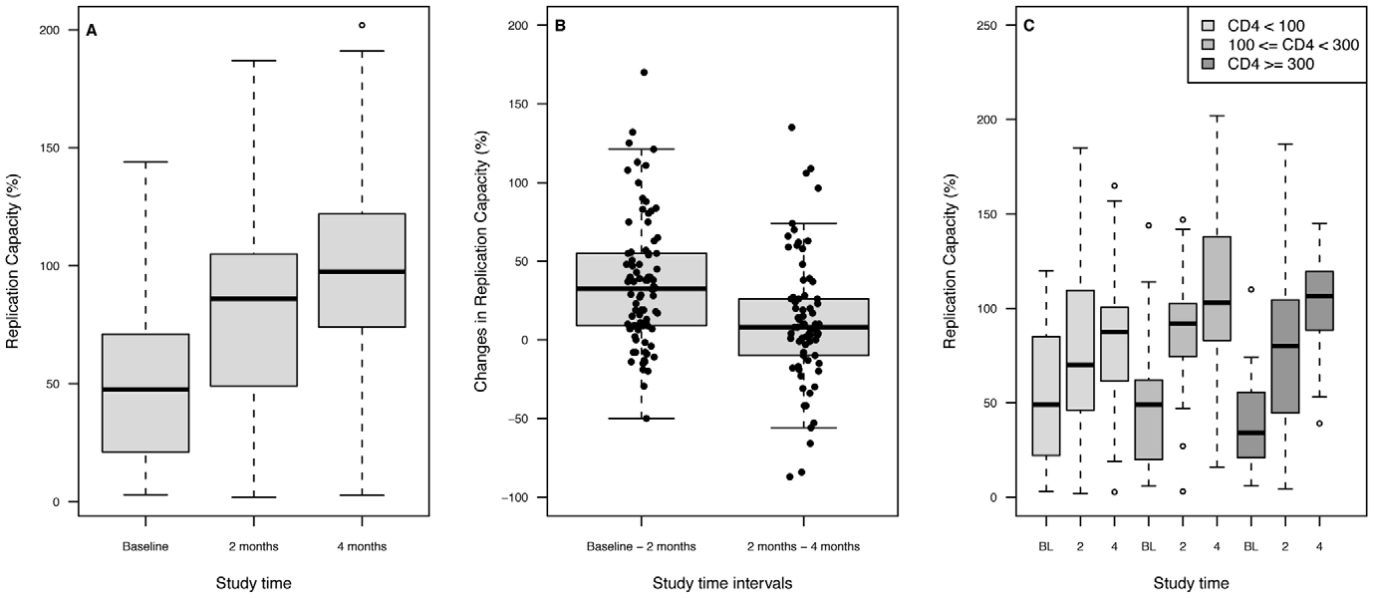
Change in Replication Capacity (RC) during 4 months of structured treatment interruption (STI). Panel A: Distribution of Replication Capacity of the patient virus at baseline (BL), 2 months and 4 months of STI. The increasing trend is significant (trend test p-value <1 10^-15^). Panel B: Distribution of RC change by time intervals (between baseline and 2 months of STI, and between 2 months and 4 months of STI). The decrease in RC changes over time shows that most changes in RC happen between baseline and 2 months. The wide range of individual values shows inter-patient variability in the reversion to wild-type. Panel C: RC change by baseline CD4 strata. The 3 groups were defined as baseline CD4 counts <100 cells/mm^3^ (lowCD4BL), 100≤CD4<300 (medCD4BL), and CD4≥300 (highCD4BL). Median RC change during 4 months of STI was 18 in lowCD4BL, 63.5 in medCD4BL and 57 in highCD4BL. The differences between ΔRC in lowCD4BL and in the 2 other groups were significant (p = 0.019 and 0.004).

Viral RC increased during the duration of STI. Between baseline and 2 months, median RC increased from 47.5% (IQR = 21.0%-70.5%) to 86.0% (49.0%-104.8%). At 4 months, median RC had increased to 97.5% (IQR = 75.3%-121.8%) (Figure 1-A). The overall positive trend was significant (p-value <2.2e-16). The increase in RC was greater during the first 2 months than during the second half of STI (p-values of 4.315e-11 and 0.01073 respectively) (Figure 1-B). It ranged from –50 to 170 between baseline and 2 months, and from –87 to 135 between 2 and 4 months.). These results were consistent with the emergence of a different viral population with greater ability to replicate in the absence of drugs over the course of the STI. The change in viral RC (ΔRC) during STI varied amongst patient samples. Few of the patients able to sustain 4 months of STI (14.4%) showed a decrease in RC during these time intervals. Inter-patient variability suggested the impact of non-viral factors; therefore we examined the effect of CD4+ cell count at baseline on subsequent viral phenotype.

Patients were grouped based on their baseline CD4: CD4<100 cells/mm^3^ (lowCD4BL, n = 37), 100≤CD4<300 (medCD4BL, n = 29), CD4≥300 (highCD4BL, n = 24). All 3 groups showed an increase in median RC (Figure 1-C). However, the extent of RC increase was significantly smaller in lowCD4BL (median ΔRC  = 18 from baseline to 4 months) than in the other groups. In medCD4BL, median ΔRC  = 63.5 (p-value = 0.019) and median ΔRC = 57 (p-value = 0.004) for highCD4BL.

### Resistance mutation prevalence and reversion rate

**Figure 2:**
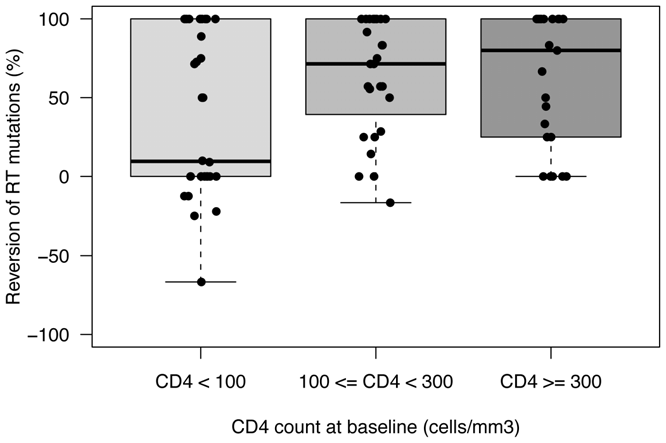
RT reversion rate by baseline CD4 strata. The reversion rate was calculated as the proportion of RT mutations reverting to wild-type codon divided by the number of RT mutations at baseline for each patient. Patients with lower CD4+ cell count at baseline (CD4<100) showed fewer reversion of RT mutations, the median proportion of RT mutations reverting for this group was 9.5%, compared to over 75% in the other groups.

All patients included in this study exhibited multiple resistance mutations at baseline with a median number of mutations of 7 and 3 for RT and PR, respectively. The number of mutations decreased to a median number of 2 RT mutations and 0 PR mutation during the STI. Over the course of the STI, we observed a shift in the distribution of the number of mutations by patient. The proportion of viruses with high number of mutations decreased progressively, while the proportion of viruses with lower number of mutations increased, indicating a continuum of mutation reversion rates. At 4 months, some patients still carried MDR viruses. To examine the impact of host factors on the ability of viruses with few mutations to grow, we analyzed the reversion rates of RT mutations within the 3 CD4 baseline groups. In lowCD4BL, we observed significantly fewer reversions than in the medCD4BL and highCD4BL groups (Figure 2). The median proportion of RT mutations reverting were 9.5% in lowCD4BL and 75% in the 2 other groups (Kruskal-Wallis rank sum test, p = 0.02).

The most prevalent mutations at baseline were M41L (73.8% of patients), M184V, and T215Y (69%, 66.7% respectively). Among those 3 mutations, M184V had the fastest rate of reversion with 40% of patients showing dominant viral strains without this mutation after 2 months of STI. In comparison, M41L and T215Y had a rate of reversion of 32% and 23% respectively within the same time period. Of all mutations studied, T215F showed the fastest reversion rate between baseline and 2 months, with 41% of the patients reversing this mutation (Table 1). Mutation reversion continued between 2 and 4 months within the STI. The rate of back-shifting of most mutations increased over time. More than 50% of the patients who still had M184V and L74V at 2 months saw an outgrowth of variants without those mutations. The rate of reversion of Y181C increased from 9% to 33%. The emergence of viral strains without the mutation slowed down for other mutations, such as L210W, which reverted in 22% of the samples between baseline and 2 months, and in 14% between 2 and 4 months. Notably, the mutation K103N had a high rate of reversion at both timepoints, with a reversion rate of 62% over the duration of the STI.

**Table 1:** Prevalence and rate of reversion of RT mutations at baseline (BL), 2 months (2mo) and 4 months (4mo) Structured Treatment Interruption (STI) and associated changes in Replication Capacity (RC).

	Prevalence baseline	Prevalence 2 months	Prevalence 4 months	% reversionBL-2mo	% reversion2mo–4mo	% reversionBL–4mo	RC gain^a^
E44D	26.2	22.6	22.6	14	5	18.2	90
T69D	21.4	16.7	14.3	22	14	33.3	76
108I	21.4	16.7	8.3	28	46	61.1	58
181C	39.3	36.9	28.6	9	33	39.4	57
184V	69.0	41.7	19	40	57	74.1	53.5
67N	57.1	42.9	31	27	26	45.8	51.25
219Q	17.9	15.5	8.3	20	50	60.0	50
118I	41.7	29.8	25	29	24	42.9	48
103N	53.6	36.9	21.4	36	41	62.2	45
215F	26.2	15.5	10.7	41	38	63.6	41
70R	22.6	21.4	14.3	32	46	63.2	39.75
41L	73.8	51.2	38.1	32	29	50.0	36
215Y	66.7	52.4	38.1	23	30	44.6	34
210W	54.8	44	36.9	22	14	32.6	30
74V	34.5	22.6	11.9	34	58	72.4	29
100I	14.3	11.9	6	17	50	58.3	16.8
190A	21.4	20.2	14.3	11	38	44.4	15.55

a : RC gain was defined as the difference in median RC between the viruses who showed the reversion of a given mutation and the viruses who kept the mutation between baseline and 2 months STI.

The prevalence of each mutation decreased over the course of the STI. The last column of the table represents the difference between the median ΔRC of the samples which reverted a mutation back to wild-type codon and the median ΔRC of the samples which did not revert the mutation. Most mutation reversions were associated with an increase in RC over the course of the STI. The largest increase in RC occurred between baseline and 2 months of STI. Between 2 months and 4 months of STI, the reversion of mutations was also associated with an increase in RC, although of a lesser magnitude since RC was already close to 100% at 2 months (data not shown).

To examine the potential genomic linkage of reversion of pairs of mutations, we calculated the frequency of pairs of RT mutations reverting concurrently between baseline and 2 months of STI. The frequency distribution ranged from 0 (no linkage) to 1 (high linkage) with a median of 0.34, 80th and 90th percentiles of 0.65 and 0.82 respectively (Table S1). The highest frequency of concurrent reversion was found for L100I and K103N. The loss of M41L was most closely associated with the loss of several mutations, including L210W (0.96), T215Y (0.91), T69D (0.89) and V118I (0.78). The mutation M184V was most likely to revert concurrently with E44D, T69D, L210W and T215Y. The reversion of mutations D67N and T215F was associated with K219Q.

### Pathway of resistance mutation reversion and change in RC

**Figure 3:**
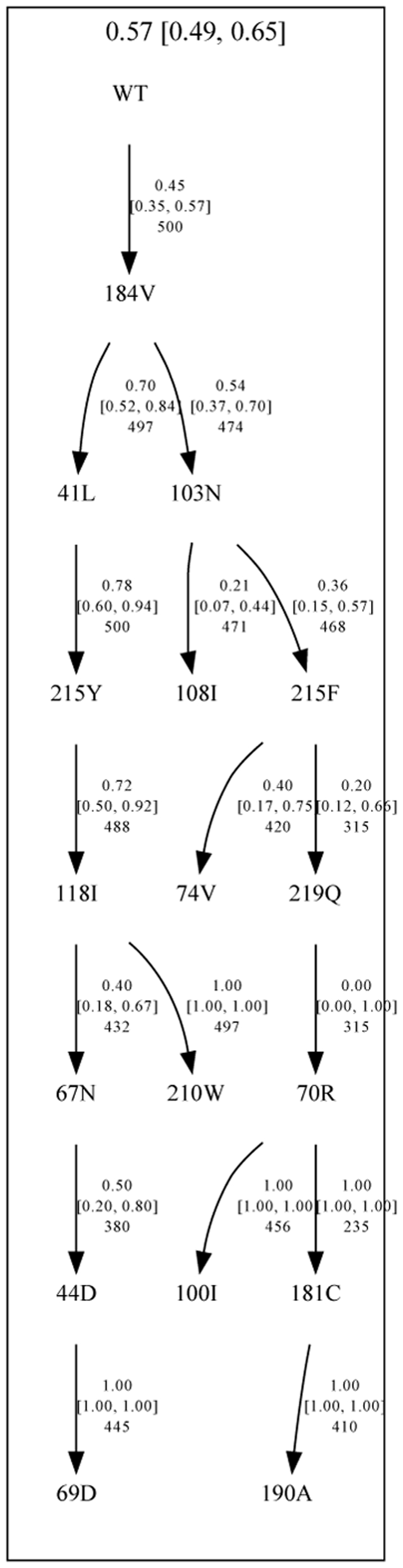
Mixture of mutagenetic trees obtained for the reversion of RT mutations. This mutagenetic tree model describes the patterns of reversion of mutations in patients with CD4+ cell count >100 at baseline. The model comprised two components: one tree representing the estimated the order of reversion of the RT mutations during the structured treatment interruption, and one star-like tree, with equal weight for each mutation, representing the noise in the model (Figure S1). The numbers at the top of each tree represent the weight of each tree component in the model, and numbers on the edges of the tree represent the conditional probability of the events.

We generated mutagenetic tree models to derive the order of reversion of RT mutations. Patients in lowCD4BL were excluded as they showed limited reversion during STI. The optimal number of trees to include in the model was selected based on 10-fold cross- validation. The resulting model comprised 2 components, a star-shaped tree representing the noise component and another tree with 2 branches (Figure 3 and Figure S1). In this latter tree, M184V was found to be the first mutation to revert. The tree separated into two branches, one branch supported by M41L, T215Y and L210W, another branch containing K103N, T215F and K219Q. The branches were supported by bootstrap analysis.

**Figure 4:**
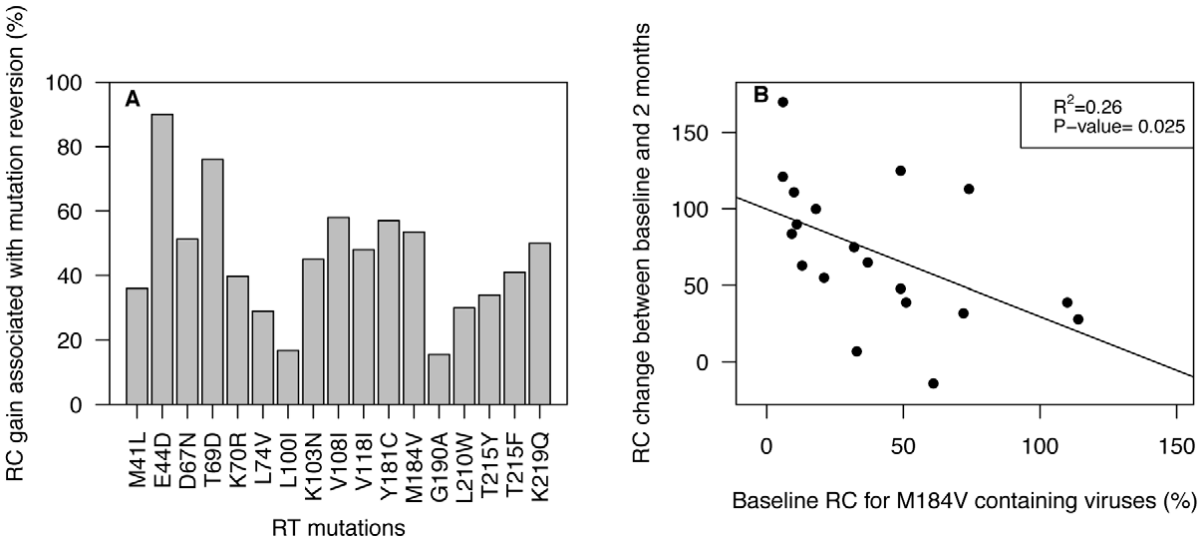
Replication Capacity (RC) gain associated with the reversion of RT resistance mutations. Panel A: Median RC gain for individual RT mutation. The RC gain is calculated as the difference in RC between the viruses which showed the reversion of a given mutation and the viruses who kept the mutation. Panel B: RC change in viruses reverting M184V to wild-type between baseline and 2 months of Structured Treatment Interruption. The RC change is calculated as the difference in RC between baseline and 2 month for each virus showing M184V at baseline. The RC change is plotted against baseline RC to show the association between baseline RC and RC change after 2 months of STI.

To examine whether the order of reversion during STI was related to an optimized benefit in viral RC, we compared the ΔRC in viruses losing or retaining a given mutation. Although all reversions were associated with increase in RC, the extent of ΔRC was variable (Table 1, Figure 4-A). Among the most prevalent mutations, reversion of M184V was associated with the largest ΔRC. Other mutations that were less frequent but associated with a large increase in RC included E44D, T69D, D67N, and 2 Non-Nucleoside RT inhibitors (NNRTI) mutations (namely V108I and Y181C). Interestingly, the potential extent of ΔRC was associated with baseline RC. Viruses with low baseline RC showed a larger spread of ΔRC than viruses with higher baseline RC values. Figure 4-B shows the association between baseline RC and RC change after 2 months of STI for the mutation M184V. Viruses that reverted M184V between baseline and 2 months of STI and with baseline RC below 50% showed a median RC change of 82% (IQR = 55% - 108.7%), while viruses with baseline RC above 50% showed a median RC change of 39.5% (IQR = 21.5% - 46.7). The correlation was significant with p-value = 0.025.

## Discussion

Antiretroviral treatment interruption is a common occurrence in clinical practice during which the restoration of viral sensitivity to antiretroviral drugs has been observed as a consequence of the outgrowth of virus strains with fewer or no resistance mutations. The present study aims to characterize the evolutionary patterns of the reversion of resistance mutations during treatment interruption. We used data collected during the duration of STI in the CPCRA 064 study for our analyses. Viral RC increased during the 4 months of STI, increasing more dramatically during the first 2 months than during the second half of the STI. The extent of RC increase was smaller in patients with low baseline CD4 cell count (CD4<100).

Our analyses showed a large variability in the pattern and rate of genotypic reversion to drug sensitivity among patients. After 4 months of STI, only 33% of the patients had reached a complete reversion to wild-type codons. The total number of mutations reverting during the STI depended on the number of mutations at baseline as well as the baseline CD4. The observation that a higher mutation reversion rate was observed in patients with higher baseline CD4 was consistent with previous findings [1], and may reflect the fact that a larger pool of wild-type integrated proviruses might be expected in this situation, representing prior circulating forms of HIV that were dominant before the institution of ART. Patients with low baseline CD4 counts showed significantly lower reversion as compared to those with higher CD4 counts, with some viruses presenting a complete (or near complete) disappearance of resistance mutations, whereas other viruses showed no change at all.

Interestingly, baseline RC was not predictive of the likelihood of reversion to wild-type HIV (data not shown). At the end of the 4 month STI, 16 of the 90 patients (17.8%) who maintained the STI for 4 months had no reversion of their mutations. This subset of 16 patients had lower CD4+ cell count at baseline (median CD4/mm^3^  = 58) and less reduction of CD4 during STI. They were also characterized by little change in viral RC and viral load (data not shown). These observations are generally characteristic of more advanced disease, possibly having longer exposure to prior ART. Once again, the lack of reversion in this subset may be explained by low levels of wild-type viruses in latently infected CD4 cells and an exhausted immunological potential for recovery. A prolonged period of STI might be required for the original wild-type virus to re-emerge in the circulating plasma viral population.

Our data analyses showed a large amount of variability in prevalence and reversion rates to wild-type codons among the resistance-associated mutations (RAMs). We observed that contrary to expectations, mutations do not appear to shift concurrently. The mutagenesis tree models showed that M184V, when present, was overall the first mutation to revert among all the RT mutations reported in this study. Our analyses suggested that the rank of M184V in the model may be explained by its high prevalence and the large increase in viral RC associated with its reversion. Moreover, there is no evidence that M184V co- reverts with any particular RT resistance mutation. This result is consistent with the findings from a recent study from Hedskog et al. where ultra-deep pyrosequencing was used to analyze in more detail the genotypic composition at specific RT positions of longitudinal samples from 6 patients [17].

Following M184V, the model of reversion divides in two branches, one consisting of M41L, T215Y and L210W (branch 1) and the other branch consisting of K103N, K219Q and other RT mutations (branch 2). In branch 1, M41L, T215Y and L210W have been shown to preferably cluster together rather than with other members of the thymidine analog resistance mutation (TAM) pathway and to have a large impact on viral fitness [18]. This may explain why they are more likely to co-revert together as well. Branch 2 grouped other TAM mutations and NNRTI resistance mutations. Interestingly, the mutation at position 103 in RT, which is not reported to revert easily [19], did revert in our study. This is most likely due to mutation linkage, since K103N had high co-reversion frequency with other mutations such as M184V and L100I.

Our results are consistent with previous observations that the loss of drug resistance mutations may occur as a consequence of emerging wild-type virus and the reversion or back mutation to a more fit state [1], [20]. Using a survival analysis model, Trignetti et al. did not find a pattern in the reversion of RT mutations during treatment interruption [21]. The difference in results may be due to the nature of the cohort data. The cohort they used was comprised of patients with varied durations of treatment interruption. We used predefined timepoints in controlled clinical settings that allow for the linking of genotype data with viral fitness at fixed time of the reversion process within the cohort.

Our analyses were performed on a subset of patients from the CPCRA 064 cohort who were able to sustain the STI for at least 4 months. However, some patients were not able to tolerate long period of treatment interruption [13]. A total of 32 patients resumed ART before the end of the 4 months STI. These patients had similar CD4+ count, RC and resistance mutations prevalence at baseline as our subset, but higher HIV RNA levels at baseline (5.4 log copies/ml vs. 4.9 log copies/ml, p<0.0001). These patients showed faster rate of reversion of RT resistance mutations during the STI compared to the subset used in our analyses (although only Y181C and G190A were significantly faster, data not shown), and a higher median RC after 2 months STI (p = 0.002), suggesting the effect of other immunological and clinical factors on viral rebound. Moreover, our analysis was limited to RT resistance mutations. More analysis, including protease inhibitors resistance mutations, is needed to fully understand the underlying mechanisms of reversion of multi-drug resistant HIV in the absence of drug pressure.

In conclusion, the CPCRA 064 study provided an opportunity to explore the genotypic changes occurring during STI and the corresponding effect on viral RC. Much inter- patient variability is observed with regard to the loss of resistance-associated mutations and the emergence of wild-type virus. The reversion of resistance mutations to wild-type codons appears to follow a predefined pattern, based on the prevalence of RAMs and mutation linkage. Using mutagenetic tree models, we found that in this dataset, M184V was the most prevalent mutation and the first to change, followed by the M41L, T215Y pathway or the K103N, K219Q pathway. These similarities between the pathways of acquisition and reversion of RAMs suggest the importance of compensatory effects between mutations. Reversion of M184V was associated with large increases in viral RC, but RC alone cannot explain that M184V reverts first, since other secondary mutations were also associated with large RC changes. The extent of viral RC increase during the STI is variable among patients, and seems to be impacted by other clinical factors, such as baseline CD4. More research is needed to understand fully the mechanisms of reversion to wild-type off therapy.

## Supporting Information

Table S1 Frequency of concurrent reversion for RT mutations. Each column of the table lists the frequency of concurrent reversion for a given mutation paired with the mutation listed in rows. The frequencies range from 0 (or NA when 2 mutations do not coexist) to 1. Mutations belonging to the same pathway of reversion appear more likely to revert at the same time.
(0.08 MB DOC)

Figure S1 Star-like mutagenetic tree obtained for the reversion of RT mutations. This tree represents the noise component in the mixture of mutagenetic trees model describing the patterns of reversion of mutations in subjects with CD4+ cell count >100 at baseline. The numbers at the top of each tree represent the weight of each tree component in the model, and numbers on the edges of the tree represent the conditional probability of the events.
(0.10 MB TIF)
