# Supplementary material for: Differences in Reversion of Resistance Mutations to Wild-Type under Structured Treatment Interruption and Related Increase in Replication Capacity
Source: PLoS One. 2011 Jan 31;6(1):e14638. doi: 10.1371/journal.pone.0014638 (PMC3031504; doi:10.1371/journal.pone.0014638)
Supplement: Table S1 — Frequency of concurrent reversion for RT mutations. Each column of the table lists the frequency of concurrent reversion for a given mutation paired with the mutation listed in rows. The frequencies range from 0 (or NA when 2 mutations do not coexist) to 1. Mutations belonging to the same pathway of reversion appear more likely to revert at the same time. (0.08 MB DOC) [file pone.0014638.s001.doc]

**Supplemental Data**

**Supplemental Table 1: Frequency of concurrent reversion for RT mutations.**

Each column of the table lists the frequency of concurrent reversion for a given mutation paired with the mutation listed in rows. The frequencies range from 0 (or NA when 2 mutations do not co-exist) to 1. Mutations belonging to the same pathway of reversion appear more likely to revert at the same time
