# Supplementary figures and images for: Differences in Reversion of Resistance Mutations to Wild-Type under Structured Treatment Interruption and Related Increase in Replication Capacity
Source: PLoS One. 2011 Jan 31;6(1):e14638. doi: 10.1371/journal.pone.0014638 (PMC3031504; doi:10.1371/journal.pone.0014638)

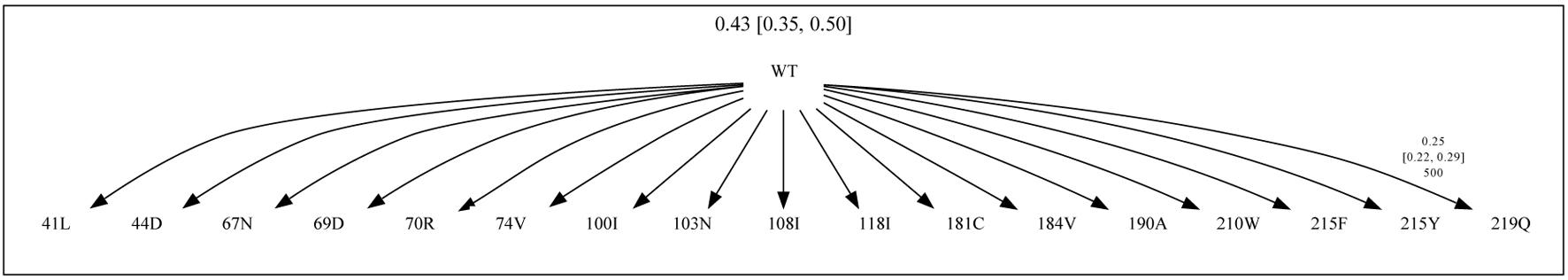

Supplement: Figure S1 — Star-like mutagenetic tree obtained for the reversion of RT mutations. This tree represents the noise component in the mixture of mutagenetic trees model describing the patterns of reversion of mutations in subjects with CD4+ cell count >100 at baseline. The numbers at the top of each tree represent the weight of each tree component in the model, and numbers on the edges of the tree represent the conditional probability of the events. (0.10 MB TIF) [file pone.0014638.s002.tif]
